# Characterization of *Cosmos sulphureus* Cav. (Asteraceae): Phytochemical Screening, Antioxidant Activity and Chromatography Analysis

**DOI:** 10.3390/plants12040896

**Published:** 2023-02-16

**Authors:** Rubí Julieta Ortega-Medrano, Luis Fernando Ceja-Torres, Monserrat Vázquez-Sánchez, Guillermo Cristian Guadalupe Martínez-Ávila, José Roberto Medina-Medrano

**Affiliations:** 1 Centro Interdisciplinario de Investigación para el Desarrollo Integral Regional Unidad Michoacán, Instituto Politécnico Nacional, Jiquilpan 59510, Michoacan, Mexico; 2 Programa de Posgrado en Botánica, Colegio de Postgraduados Campus Montecillo, Texcoco 56264, Estado de Mexico, Mexico; 3 Laboratorio de Química y Bioquímica, Facultad de Agronomía, Universidad Autónoma de Nuevo León, General Francisco Villa S/N, Ex-Hacienda “El Canadá”, General Escobedo 66050, Nuevo Leon, Mexico; 4 Licenciatura en Genómica Alimentaria, Universidad de La Ciénega del Estado de Michoacán de Ocampo, Sahuayo 59103, Michoacan, Mexico

**Keywords:** phenolic compounds, high-performance thin-layer chromatography (HPTLC), rutin, quercetin

## Abstract

*Cosmos sulphureus* Cav. (Asteraceae), and endemic plant of Mexico is used in herbal medicine. In this study, the phytochemical composition, phenolic content, and antioxidant activity of ethanolic and methanolic extracts from *C. sulphureus* leaves and flowers were determined. The phytochemical analysis showed the presence of compounds such as terpenoids, phenolic compounds, tannins, and flavonoids and the absence of alkaloids, saponins, glycosides, and anthraquinones. The experimental results showed that the extracts have high contents of phenolic, flavonoid, and condensed tannins contents. The phenolic compounds identified in the *C. sulphureus* extracts by high-performance thin-layer chromatography (HPTLC) include phenolic acids such as chlorogenic acid and caffeic acid as well flavonoids such as rutin and quercetin. The *C. sulphureus* extracts showed a relevant free radical scavenging activity, ferric-reducing antioxidant power, lipid peroxidation inhibition ability, and oxygen radical antioxidant capacity. This research highlights the phytochemical profile and antioxidant activity of phenolic compounds-rich extracts from *C. sulphureus* leaves and flowers.

## 1. Introduction

Mexico is considered the center of diversification of several groups of plants [1], among which the Asteraceae (Compositae) family stands out. In some studies that have been carried out on the diversity of plants in Mexico, it is mentioned that this family represents one of the most diverse, currently with 417 genera and 3113 species, which are widely distributed throughout the national territory, of which 3050 correspond to native species and 1988 to endemic species [2]. Their wide geographical distribution and richness of species are attributed to their great dispersal capacity. In addition, they have a wide genetic plasticity and a great capacity to adapt to different climatic conditions due to the morphological and genotypic variability that they present, and that allows them to have different reproduction strategies and forms of growth [3,4,5,6].

The genus *Cosmos* belongs to the Asteraceae family and is made up of 33 species and four varieties that can be found in Mexico [7]. Some species that belong to this genus are cultivated as ornamental plants, and others are known as ruderal weeds. Their distribution extends from the United States of America to northern Argentina [8,9].

The species *Cosmos sulphureus* Cav. is a native plant in Mexico with a wide distribution in America, being widely found in Mexico, as well as in the Caribbean islands, Central America, and South America [8]. It is a short-lived annual wild plant that can reach 2 m in height, and its flowering occurs between the months of August to December. It can be found in areas of secondary vegetation derived from the tropical deciduous forest [10]. *C. sulphureus* has edible flowers with a vibrant yellow or orange color, which are widely used in gourmet cuisine as an ingredient in salads and as a garnish in cakes or savory dishes for their slightly bitter and spicy flavor [11]. The inflorescences of *C. sulphureus* have been considered important sources of yellow to orange dyes since pre-Columbian civilizations from Central and South America [12]. In Mexico, it is known as “girasol” or “mirasol amarillo” and is used in traditional medicine [13]. Currently, *C. sulphureus* has been analyzed as a possible source of antioxidant compounds with biological activity.

The aim of the present study was to determine the phytochemical composition, phenolic content, and antioxidant activity of ethanolic and methanolic extracts from *C. sulphureus* leaves and flowers.

## 2. Materials and Methods

### 2.1. Chemicals and Reagents

The hydrochloric acid (HCl), lead acetate, ferric chloride, ammonium hydroxide, sodium hydroxide (NaOH), chloroform, sulfuric acid (H_2_SO_4_), Barfoed’s reagent, Felhing’s solution A and B, copper sulfate, ninhydrin, acetone, acetic anhydride, Folin–Ciocalteu reagent, 2,2-diphenyl-1-picrylhydrazyl radical (DPPH^•^), sodium carbonate, aluminum chloride (AlCl_3_), sodium nitrite (NaNO_2_), ethyl acetate, formic acid, acetic acid, 2-aminoethyl diphenyl borate, potassium ferrocyanide, trichloroacetic acid, ferric chloride, linoleic acid, Tween 20, ferrous chloride, ethylenediaminetetraacetic acid (EDTA), fluorescein, 2,2′-azo-bis(2-amidino-propane)dihydrochloride (AAPH), trolox, ethanol, methanol, and water (HPLC grade) were purchased from Sigma-Aldrich (St. Louis, MO, USA). Plates 20 × 10 cm HPTLC Silica gel 60 F_254_ were purchased from Merck (Kenilworth, NJ, USA).

### 2.2. Standards

Caffeic acid, chlorogenic acid, ferulic acid, gallic acid, *p*-coumaric acid, cinnamic acid, catechin, naringin, apigenin, rutin, quercetin, and vanillin (>99%) were purchased from Sigma-Aldrich (St. Louis, MO, USA). The stock solutions were prepared by diluting the standards in methanol (5 mg/mL). The stock solutions were then diluted in methanol to obtain working solutions (0.5–3 mg/mL). Working solutions of standards were stored at –20 °C in dark conditions.

### 2.3. Plant Material

*C. sulphureus* specimens were collected during September and October 2017 at Jiquilpan (19°58.728′0″ N, 102°42.698′0″ W) and Sahuayo (20°1.528′0″ N, 102°43.340′0″ W), in the Mexican state of Michoacán. Three populations were collected, namely, Population 1 (P1), Population 2 (P2), and Population 3 (P3). The specimens were authenticated by one of the authors, Dr. Monserrat Vázquez Sánchez. Voucher herbarium specimens were deposited at the herbarium of Centro Interdisciplinario de Investigación para el Desarrollo Integral Regional Unidad Michoacán, with herbarium numbers 11578 and 11579.

### 2.4. Drying Process

Foliar (leaf) and reproductive tissues (flower) were removed from the *C. sulphureus* plants and dried in a horizontal airflow oven model TE-FH45DM (Terlab, Zapopan, Mexico) at 50 °C for 24 h. Afterward, the tissues were ground in a blender to obtain a fine powder. Additionally, to homogenize the particle size to 250 µm, a sieve (number 60) was used. Milled tissues were stored in dark conditions at room temperature until they were used.

### 2.5. Preparation of Extracts

The extraction of phenolic compounds was achieved using 3 g of dry-milled plant materials, which were suspended in 40 mL of solvent (70% ethanol, and 70% methanol, *v*/*v*) by agitation at 100 rpm using an orbital shaker apparatus, model Sea Star (Heathrow Scientific, Vernon Hills, IL, USA), in the dark at room temperature for 24 h. Then, the extracts were centrifuged at 2722× *g* for 10 min at room temperature. The supernatant was recovered in new tubes, and again, the precipitate was shaken with 20 mL of the corresponding solvent at 100 rpm for 3 h. Subsequently, they were centrifuged at 2722× *g* for 5 min, and the supernatant was recovered again to obtain a final volume of extract of 60 mL. Both supernatants were combined to form the crude extract. Finally, the extract was filtered through Whatman No. 1 filter paper (Whatman International Ltd., Maidstone, UK). Aliquots of the crude extract were taken for the phytochemical screening.

### 2.6. Qualitative Analysis of Phytochemical Screening

In order to determine the presence of chemical compounds in the crude extracts of *C. sulphureus*, the following determinations [14,15] with some modifications were used:

#### 2.6.1. Determination of the Presence of Alkaloids Using the Wagner Test

1.5 mL of 2 M HCl was combined with 1.5 mL of *C. sulphureus* extract, then a few drops of Wagner’s reagent were added and shaken for a few seconds. The reddish-brown color change and a precipitate indicated the presence of alkaloids.

#### 2.6.2. Determination of Phenolic Compounds Using the Lead Acetate Test

1.5 mL of extract was combined with 2.5 mL of distilled water, and 1.5 mL of 10% lead acetate solution was added. To confirm the presence of phenolic compounds, the formation of a voluminous white precipitate was visible.

#### 2.6.3. Determination of the Presence of Tannins Using the Ferric Chloride Test

A few drops of a 5% ferric chloride solution were added to 1.5 mL of the *C. sulphureus* extract. The color change of the extract to green-blue indicated the presence of tannins.

#### 2.6.4. Determination of the Presence of Flavonoids by Ammonium Hydroxide Test

1.5 mL of each extract was taken and combined with 1.5 mL of 5% ammonium hydroxide. The color change to fluorescent yellow indicated the presence of flavonoids.

#### 2.6.5. Determination of the Presence of Glycosides Using the Bornträger Test

To 1.5 mL of each extract, 3 mL of chloroform was added, shaken, and the chloroform layer was recovered, which was combined with 2 mL of a 10% ammonia solution. The color changed to pink in the solution, indicating the presence of glycosides.

#### 2.6.6. Salkowski Test to Determine the Presence of Sterols

To 1.5 mL of each extract, 1.5 mL of chloroform and 1.5 mL of concentrated H_2_SO_4_ were added and stirred for 5 min. If the chloroform layer was seen to change to red color, and if the formation of the acid layer showed fluorescent yellow-green color, then the presence of sterols was confirmed.

#### 2.6.7. Determination of the Presence of Terpenoids

To 1.5 mL of each extract, 2 mL of concentrated chloroform was added, the samples were shaken for a few seconds, and 1 mL of concentrated sulfuric acid was added. They were shaken manually, and the changes in each sample were observed. The presence of terpenoids was indicated by the color change to reddish brown.

#### 2.6.8. Barfoed Test to Determine the Presence of Carbohydrates

1.5 mL of Barfoed’s reagent was added to 1.5 mL of extract, mixed, then boiled in a water bath for 2 min. The color change of the precipitate to red indicated the presence of carbohydrates.

#### 2.6.9. Fehling’s Test to Determine the Presence of Carbohydrates

To 500 µL of each *C. sulphureus* extract, 500 µL of Felhing’s solutions A and B were added. The color change was observed, which, if it is red, will indicate the presence of carbohydrates.

#### 2.6.10. Biuret Test for the Determination of the Presence of Proteins

1.5 mL of the extract was taken, and a drop of 2% copper sulfate solution was added. Then, 1.5 mL of 95% solvent (ethanol, methanol) was added, followed by potassium hydroxide. The pink coloration formed in the solvent layer will indicate the presence of proteins.

#### 2.6.11. Ninhydrin Test for the Determination of the Presence of Amino Acids

2 drops of ninhydrin solution (5 mg in 200 mL acetone) were added to 1.5 mL of extract. The characteristic purple color formation indicates the presence of amino acids.

#### 2.6.12. Determination of the Presence of Saponins

To 1.5 mL of each extract, 2 mL of distilled water was added. It was then vortexed for 15 min. The formation of 1 cm foam indicated the presence of saponins.

#### 2.6.13. Determination of the Presence of Coumarins

To 1.5 mL of each *C. sulphureus* extract, 1.5 mL of 10% NaOH was added. A color change to yellow indicated the presence of coumarins.

#### 2.6.14. Determination of the Presence of Quinones

1.5 mL of sulfuric acid was added to 1.5 mL of each extract. The color change to red indicated the presence of quinones.

#### 2.6.15. Determination of the Presence of Anthraquinones

A few drops of 2% HCl were added to 1.5 mL of extract. The color change to red indicated the presence of anthraquinones.

#### 2.6.16. Determination of the Presence of Sterols

1.5 mL of extract was mixed with 1.5 mL of chloroform, then 1.5 mL of acetic anhydride and 2 drops of concentrated H_2_SO_4_ were added and placed on the walls of the test tube. The color change to red, followed by blue and then green, indicating the presence of sterols.

### 2.7. Sample Preparation for Phenolic Content, Antioxidant Activity, and High-Performance Thin-Layer Chromatography (HPTLC) Analysis

Prior to analysis, the extracts (15 mL) were lyophilized in a Labconco FreeZone freeze dryer (Labconco, Kansas City, MO, USA) at −55 °C for 24 h and under high vacuum conditions (0.02 mBar). After this, 20 mg of each extract was resuspended individually in 1 mL of solvent (70% ethanol, and 70% methanol, *v*/*v*). Finally, the extracts were stored under refrigeration at 4 °C, protected from light.

### 2.8. Determination of Total Phenolic, Flavonoid, and Tannin Contents

#### 2.8.1. Total Phenolic Content

The determination of the total phenolic content of the extracts was carried out according to Castro-López et al. [16] with modifications. Twenty-five µL of the *C. sulphureus* extract was placed in a 96-well plate and mixed with 25 µL of 1 N Folin–Ciocalteu reagent. The plate was placed in a Synergy HTX Multi-Mode microplate reader (Biotek, Rochester, VT, USA). The microplate reader placed 25 µL of 20% sodium carbonate, homogenized and incubated for 30 min, then the dispenser placed 200 µL of distilled water for each sample and subsequently read the absorbance at 750 nm. Data were acquired using Gen5 Data Analysis Software version 2.06 (Biotek, Rochester, VT, USA). Total phenolic contents were estimated using a gallic acid standard curve (A_760_ = 5.0271 [gallic acid] −0.0512, *R*^2^ = 0.9663) obtained using six known concentrations (20–200 µg/mL) of gallic acid. The total phenolic content was expressed as milligrams of gallic acid equivalents per gram of dry extract (mg GAE/g DE).

#### 2.8.2. Total Flavonoid Content

The total flavonoid content of the extracts was determined by the aluminum chloride method previously reported [16]. Fifteen microliters of the extract were added with 15 µL of 5% (*w*/*v*) sodium nitrite (NaNO_2_). Then, 15 µL of 10% aluminum chloride (AlCl_3_) and 100 µL of 1 M sodium hydroxide (NaOH) were added. Absorbance was measured after 10 min at 510 nm using a Synergy HTX Multi-Mode microplate reader (Biotek, Rochester, VT, USA). Total flavonoid contents were calculated using a quercetin standard curve (A_510_ = 2.1144 [quercetin] −0.0177, *R*^2^ = 0.9955) obtained using seven concentrations of quercetin (5–100 µg/mL). Flavonoid contents were expressed as milligrams of quercetin equivalents per gram of dry extract (mg QE/g DE).

#### 2.8.3. Determination of Condensed Tannin Content

In order to determine procyanidin contents in the extracts, the vanillin-H_2_SO_4_ methodology was conducted with some modifications [17]. Two hundred and fifty µL of the extract was reacted with 250 µL of 1% vanillin (*w*/*v*, dissolved in methanol), followed by 250 µL of the 25% sulfuric acid solution (*v*/*v*, dissolved in methanol) and incubated at a temperature of 30 °C for 15 min. Absorbance was read at 500 nm using a Synergy HTX Multi-Mode microplate reader (Biotek, Rochester, VT, USA). Total tannin contents were estimated using a calibration curve with catechin (A_500_ = 1.9461 (catechin) −0.0007, *R*^2^ = 0.9990) using nine concentrations of catechin (10–100 µg/mL). Condensed tannin contents were expressed as milligrams of catechin equivalents per gram of dry extract (mg CE/g DE).

### 2.9. Identification and Quantification of Phenolic Compounds by High-Performance Thin-Layer Chromatography (HPTLC)

The determination and quantification of phenolic compounds in the extracts of leaves and flowers of *C. sulphureus* was carried out following the methodology established by Stanek & Jasicka-Misiak [18] with some modifications. A Camag HPTLC instrumental set-up (Camag, Muttenz, Switzerland) consisting of an Automatic Sample Applicator (ATS 4), an Automatic Developing Chamber (ADC 2), a Chromatogram Immersion Device III, a TLC Plate Heater III, a TLC Visualizer, and VisionCats version 2.5.18072.1 data processing software was used for the analysis. Standard and extract solutions (5 µL) were applied bandwise (band length 8 mm, 15 nL/s delivery speed under nitrogen 6 bars pressure, track distance 9.4 mm, and distance from the edge 20.1 mm) on an HPTLC glass plate (20 × 10 cm) coated with 200 µm layer thickness of silica gel 60 F_254_ by an Automatic TLC Sampler (ATS 4). Plates were developed in an Automatic Developing Chamber (ADC 2) using two different mobile phases: the first one consisted of chloroform, ethyl acetate, and formic acid (5:4:1, *v*/*v*/*v*); the second one in ethyl acetate/formic acid/acetic acid/water (100:11:11:26, *v*/*v*/*v*/*v*) over a distance of 70 mm from the lower edge of the plate. The saturation time of the chamber was conditioned and optimized to 5 min at room temperature (22 °C ± 2) and relative humidity (33 ± 2%) for better resolution with mobile phase vapors. The plates were dipped in 200 mL of a solution of 1% 2-aminoethyl diphenyl borate (*w*/*v*) using a Chromatogram Immersion Device IIP (speed 50 mm/s, time 1 s), and subsequently heated (90 °C, 3 min) in a TLC Plate Heater III. The chromatograms were documented under visible and UV (λ_max_ 254 and 366 nm) light after development and after post-chromatographic derivatization with the use of a TLC Visualizer 2 and a computer program, VisionCats version 2.5.18072.1.

Phenolic compounds were identified in the derivatized plates (with 1% 2-aminoethyl diphenyl borate solution) based on the band colors as well as retardation factors (R_f_) of the standard in comparison with constituents of the analyzed extracts. The standards used were: caffeic acid, chlorogenic acid, ferulic acid, gallic acid, *p*-coumaric acid, cinnamic acid, catechin, naringin, apigenin, rutin, quercetin, and vanillin. The quantification of each phenolic compound was accomplished based on the regression equation of each concentration peak area plotted against the concentration of phenolic compound spotted (0.5, 1.0, 1.5, 2.0, 2.5, and 3.0 mg/mL), and the results were expressed as micrograms per milliliter of extract (µg/mL).

### 2.10. Antioxidant Activity Assays

#### 2.10.1. DPPH^•^ Antioxidant Assay

The determination of free radical scavenging activity was performed using the DPPH^•^ method previously described with modifications [19]. Firstly, a 24 µM ethanol solution of DPPH^•^ was prepared. Then, 900 µL of DPPH^•^ reagent previously prepared was mixed with 100 µL of extract (0.1–1 mg/mL), and they were incubated for 10 min at room temperature. After incubation, the absorbance was measured at 523 nm using a PowerWave HT Microplate Spectrophotometer (BioTek Instruments, Inc., Winooski, VT, USA). The scavenging effect of DPPH^•^ was measured using the formula:

DPPH^•^ scavenging effect (%) = [*A*_control_ − *A*_sample_/*A*_control_] × 100 (1)

where *A*_control_ is the absorbance of the control (DPPH^•^ solution), and *A*_sample_ is the absorbance of the test sample (DPPH^•^ solution plus 100 µL of extract). The median inhibitory concentration (IC_50_) was determined using linear regression. The scavenging activity was expressed as the IC_50_ that represents the *C. sulphureus* extract concentration (mg/mL) needed to reduce by 50% the initial DPPH^•^ absorbance.

#### 2.10.2. Ferric Reducing Power (FRP) Assay

The capacity of *C. sulphureus* extracts to reduce Fe^3+^ was assessed by the FRP method [16]. In order to achieve this, 5 µL of the extract was combined with 12 µL of phosphate buffer (1 M, pH 7) and 22 µL of 1% potassium ferrocyanide. Samples were incubated at 50 °C for 15 min. After incubation, 12 µL of 10% trichloroacetic acid, 45 µL of distilled water, and 10 µL of 0.1% ferric chloride were added. Absorbance was measured at 700 nm using a Synergy HTX Multi-Mode microplate reader (Biotek, Rochester, VT, USA). Results were expressed as the median inhibitory concentration (IC_50_), which represents the extract concentration (mg/mL) required to reduce by 50% the initial absorbance.

#### 2.10.3. Lipid Peroxidation Inhibition Assay

The lipid peroxidation inhibition ability of the extracts was carried out following the methodology established by Castro-López et al. [16]. First, 1500 µL of acetic acid buffer solution (0.02 M, pH 4) was combined with 50 µL of the *C. sulphureus* extracts. Then, 100 µL of linoleic acid solution (0.6 g of linoleic acid, 1.5 g of Tween 20, 8 mL of ethanol) was added. Samples were homogenized and incubated in a water bath at 37 °C for 2 min. Subsequently, 750 µL of a 50 mM ferrous chloride (Fe_2_Cl_2_) solution containing 17 mg EDTA was added to induce the lipid oxidation and incubated for 24 h at 37 °C. Two aliquots (250 µL) were withdrawn during this period, at 0 and 24 h. After incubation, 1 mL of 10 mM sodium hydroxide solution was added to stop the oxidation process. Afterward, 2.5 mL of 10% ethanol was added. Finally, the samples were homogenized, and the absorbance was measured at 232 nm using a SmartSpec™ Plus Spectrophotometer (Bio-Rad Laboratories, Hercules, CA, USA). Phosphate buffer (pH 7) was used as blank. The percentage inhibition of linoleic acid oxidation was calculated using the following equation:

Lipid peroxidation inhibition (%) = [*A*_control_ − *A*_sample_/*A*_control_] × 100 (2)

where *A*_control_ is the difference between the absorbance of the control (phosphate buffer) after 0 and 24 h of incubation, and *A*_sample_ is the difference between the absorbance of each extracted sample after 0 and 24 h of incubation. Results were expressed as a percentage of lipid oxidation inhibition.

#### 2.10.4. Oxygen Radical Absorbance Capacity (ORAC) Assay

In order to determine the oxygen radical antioxidant capacity of the extracts, the methodology described by Huang et al. [20] was used. For this, 150 µL of 81 µM fluorescein was mixed with 25 µL of the *C. sulphureus* extract (0.5 mg/mL) and incubated at 37 °C for 30 min. Then, 25 µL of 2,2′-azo-bis(2-amidino-propane)dihydrochloride (AAPH, 153 mM, dissolved in phosphate buffer pH 7.4) was added, and the mixture was shaken for 10 s. Absorbance was recorded every 2 min for 2 h (excitation at 485 nm, emission at 528 nm) using a Synergy HTX Multi-Mode microplate reader (Biotek, Rochester, VT, USA). A calibration curve was made using Trolox at different concentrations (6.25, 12.5, 25, 50, and 100 µM). A blank (phosphate buffer pH 7) was used instead of the extract. To calculate the total area under the curve (AUC), the following equation was used:

AUC = (1 + RFU_1_/RFU_0_ + RFU_2_/RFU_0_ + RFU_3_/RFU_0_… + RFU_59_/RFU_0_ + RFU_60_/RFU_0_ (3)

where RFU_0_ corresponds to the relative fluorescence value of time zero and RFUx = represents the relative fluorescence value of time points (e.g., RFU_3_ is the relative fluorescence value at time three). Finally, Net AUC was then calculated by subtracting the Blank AUC from the AUC of each sample using the equation defined in [21]:

Net AUC = AUC_Trolox_ − AUC_blanck_ (4)

Results were expressed as µM Trolox equivalents (TE).

### 2.11. Statistical Analysis

Results were reported as mean ± standard deviation of three independent replicates. An analysis of variance (ANOVA) was used to assess statistical significance. Differences between values with a *p* < 0.05 were considered statistically significant. Tukey’s test was performed for the comparison of means for the corresponding results. Relationships between all determinations were tested using Pearson’s correlation. These analyses were performed with the SPSS software version 25.0 (SPSS Inc., Chicago, IL, USA).

## 3. Results and Discussion

### 3.1. Phytochemical Screening

The qualitative phytochemical analysis of the methanolic and ethanolic extracts of the leaf and flower of *C. sulphureus* from the three populations analyzed revealed the presence of phenolics, flavonoids, tannins, terpenoids, and carbohydrates (Table 1). Regarding the coumarin compounds, they were only present in the leaf tissues, while the quinone and sterol compounds were present in the flower tissue of the three populations, according to Table 1. The presence of alkaloids, glycosides, proteins, amino acids, saponins, and anthraquinones was not detected. The three populations showed the same behavior.

The above results are consistent with those reported by Jadav & Gowda [22], who reported the presence of compounds such as phenolics, flavonoids, tannins, and fats and oils, and the absence of carbohydrates, proteins, amino acids, steroids, glycosides, saponins, coumarins, and alkaloids in methanolic extracts of flowers from *C. sulphureus*. However, in another investigation also carried out on methanolic extracts of *C. sulphureus*, practically all the compounds determined in this investigation were positive [23]. The variability of finding one compound or another present is directly influenced by the extraction method, the solvent used in the extraction, the tissue analyzed, the phenological state of the plant, as well as the climatic conditions, among many other factors. While in our research, we analyzed the tissues separately (leaf and flower), in the previously mentioned work, the entire plant was analyzed.

### 3.2. Total Phenolic, Flavonoids, and Tannins Contents

The total phenolic contents of the extracts from *C. sulphureus* leaves, and flowers ranged from 40.74 ± 2.34 to 124.11 ± 7.77 mg GAE/g DE (Table 2). The highest phenolic content was observed in flowers from Population 1 extracted with 70% methanol (P1-F-M), and the lowest was observed in flowers from Population 2 extracted with 70% methanol (P2-F-M). The mean total phenolic content value of the extracts of *C. sulphureus* analyzed in this study was 80.29 mg GAE/g DE.

Previous studies reported total phenolic contents of 13.08 mg GAE/g fresh weight (FW) in ethanolic extracts from *C. sulphureus* flowers [24], while methanolic extracts from flowers reported 15 [25] and 86.8 [26] mg GAE/g dry weight (DW). The highest reported concentration is 102.5 mg GAE/g DW in *C. sulphureus* petals extracted with acidified ethanol [27]. The contents mentioned above represent the concentration of total phenolics present in each gram of plant tissue. In our research, the total phenolic content represents the concentration present in each gram of dry extract. In another investigation that reports in a similar way to that of our work, contents of 3.14 mg GAE/g extract were obtained in methanolic extracts from *C. sulphureus* leaves [28], with lower values compared to those obtained from the extracts analyzed here.

In the case of total flavonoid content, significant differences were detected between treatments, according to Table 2. The highest content was observed in flower extract from Population 2 extracted with 70% methanol (P2-F-M), with 162.55 ± 9.32 mg QE/g DE, while flower extract from Population 3 extracted with 70% ethanol (P3-F-E) showed the lowest flavonoid content, with 50.16 ± 7.65 mg QE/g DE. The concentration of the total flavonoid content in the methanolic extracts from *C. sulphureus* leaves, and flowers was higher compared to previously published data (2.04 mg rutin equivalents (RE)/g extract for methanolic extracts from *C. sulphureus* leaves and 57.0 mg RE/g DW for methanolic extracts from *C. sulphureus* flowers) [26,28].

Regarding the condensed tannin content, as seen in Table 2, the lowest contents were observed in flower extracts (both solvents), while the highest contents were observed in leaf extracts from Population 3 extracted with both solvents 70% ethanol and 70% methanol (P3-L-E and P3-L-M), with 60.99 and 68.70 and mg CE/g DE, respectively. Previous research results have shown a concentration of 2.03 ± 0.05 mg tannic acid equivalents (TAE)/g extract [28], lower values compared to those obtained by extracts of *C. sulphureus* analyzed in this study.

### 3.3. Identification and Quantification of Phenolic Compounds by HPTLC

**Figure 1:**
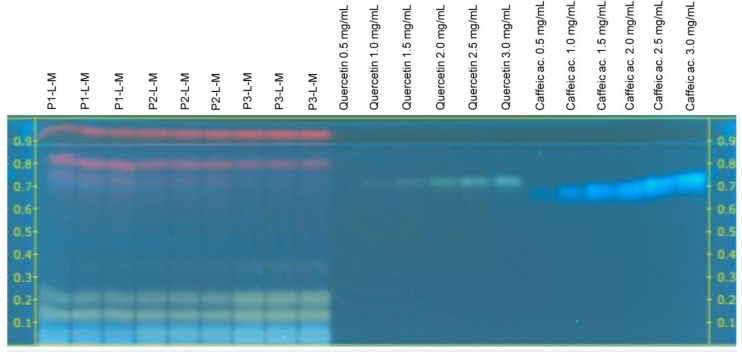
HPTLC chromatogram of methanolic extracts from leaves of *Cosmos sulphureus* under visible light in the transmittance mode developed using ethyl acetate (100): formic acid (11): acetic acid (11): water (26) (*v*/*v*/*v*/*v*) as the mobile phase after derivatization with 1% 2-aminoethyl diphenyl borate (*w*/*v*).

**Figure 2:**
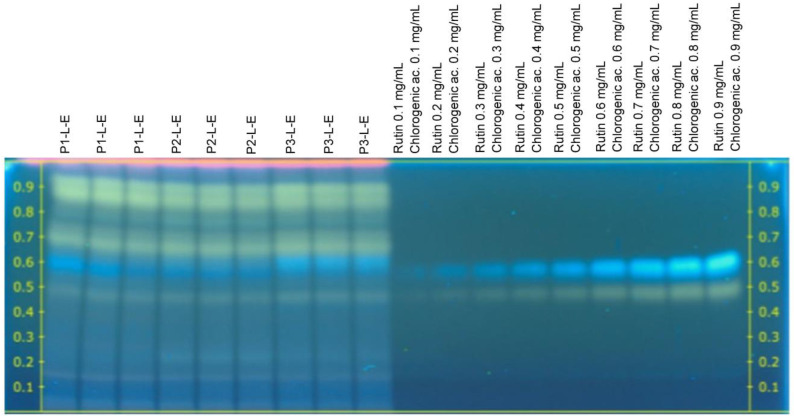
HPTLC chromatogram of ethanolic extracts from leaves of *Cosmos sulphureus* under visible light in the transmittance mode developed using ethyl acetate (100): formic acid (11): acetic acid (11): water (26) (*v*/*v*/*v*/*v*) as the mobile phase after derivatization with 1% 2-aminoethyl diphenyl borate (*w*/*v*).

Derivatization with a 2-aminoethyl diphenyl borate reagent was used to detect the presence of phenolic compounds in the *C. sulphureus* extracts. The 2-aminoethyl diphenyl borate reagent reacts with phenolic compounds to form differently colored zones. The analysis by HPTLC showed blue, yellow, pink, violet, and green-colored bands in the plates derivatized with 2-aminoethyl diphenyl borate reagent (Figure 1 and Figure 2).

According to Figure 1 and Figure 2, rutin (yellow) and chlorogenic acid (blue) bands were observed in all the extracts from *C. sulphureus* analyzed in this study (R_f_ 0.48 ± 0.04 and 0.60 ± 0.04, respectively), while in the case of caffeic acid (blue) and quercetin (green) bands, they were only observed in the *C. sulphureus* leaf extracts (R_f_ 0.71 ± 0.03 and 0.78 ± 0.04, respectively). The pink and purple-colored bands were not identified as phenolic compounds according to the standards used for the analysis.

The analysis by HPTLC showed the presence of two phenolic acids: chlorogenic acid (**2**) and caffeic acid (**3**), and two compounds belonging to the group of flavonoids: rutin (**1**) and quercetin (**4**), in the extracts of *C. sulphureus*, while the compounds ferulic acid, gallic acid, *p*-coumaric acid, cinnamic acid, catechin, naringin, apigenin, and vanillin were not identified. The concentrations of these compounds varied between tissues and solvents, as seen in Table 3 and Table 4. The phenolic composition of *C. sulphureus* samples was in agreement with previously published data [23,26,29].

Regarding phenolic acids, the highest concentration of chlorogenic acid (**2**) occurred in flower extract from Population 1 extracted with 70% methanol, with 27.51 µg/mL (Table 3), while for caffeic acid, the highest concentration was observed in flower extract from Population 1 extracted with 70% ethanol, with 175.93 µg/mL (Table 4). Regarding flavonoids, the highest concentration of rutin (**1**) was observed in flower extract from Population 2 extracted with 70% ethanol, with 27.93 µg/mL (Table 4), while for quercetin (**4**), the highest concentration was observed in flower extract from Population 1 extracted with 70% ethanol (330.70 µg/mL). The correlation analysis indicates a significant positive correlation between condensed tannin content and rutin content (*R* = 0.783, *p* ˂ 0.01), which indicates that by increasing the content of tannins in the extracts, the rutin content increased. The quantification of caffeic acid (**3**) and quercetin (**4**) in the leaf tissues of the three populations, extracted with both solvents, was not achieved due to the low concentration they presented.

Of the four phenolic compounds identified, quercetin (**4**) was the major constituent in the *C. sulphureus* flower extracts. This compound is one of the flavonoids mostly found in vegetables, recognized for its potent antioxidant activity both in vitro and in vivo. In addition, it has many biological applications [30]. Quercetin is conditioned by the location of the compound in the plant because the highest concentration of this compound is present in the external parts of the plant [31]. In the present study, a higher concentration of this compound was found in flower tissues. On the other hand, caffeic acid is a metabolite of chlorogenic acid, so the presence of chlorogenic acid may predispose to the creation of hydroxycinnamic acid derivatives such as caffeic acid [32]. In this work, the presence of caffeic acid was not determined in the *C. sulphureus* leaf extracts, although chlorogenic acid was identified.

### 3.4. Antioxidant Activity

In this study, we investigated the in vitro free radical scavenging activity, ferric-reducing antioxidant power, lipid peroxidation inhibition ability, and oxygen radical antioxidant capacity of *C. sulphureus* extracts. As seen in Table 2, a significant difference between the antioxidant profiles of the extracts was observed.

According to the analysis, the extract P1-L-M, obtained from leaves of Population 1 with 70% methanol, exhibited the strongest DPPH^•^ radical scavenging activity with an IC_50_ value of 0.196 ± 0.03 mg/mL (Table 2). Previous studies reported an IC_50_ of 135.53 ± 1.78 μg/mL in aqueous leaf extract [33] and an IC_50_ of 129.21 μg/mL in ethyl acetate fractions from *C. sulphureus* [34]. The concentration required to inhibit the DPPH^•^ radical shown by the extracts of *C. sulphureus* was similar to that previously reported.

Regarding the ferric reducing capacity of the leaf and flower extracts of the wild populations of *C. sulphureus*, the highest reducing capacity of the Fe^3+^ ion was obtained by the extracts P2-F-E and P1-F-M with an IC_50_ of 0.30 and 0.31 mg/mL, respectively, while the extract P3-L-M showed the lowest ferric reducing capacity, with as an IC_50_ of 0.74 mg/mL, as observed in Table 2. The correlation between the condensed tannin content and the ferric-reducing capacity of the extracts was shown to be the highest correlation between the different determinations carried out (*R* = 0.898, *p* ˂ 0.01). Therefore, the capacity of *C. sulphureus* extracts to reduce Fe^3+^ ion can be attributed to the condensed tannin content.

As can be seen in Table 2, the highest percentage of inhibition of the lipid peroxidation of linoleic acid that the extracts showed was 83.83 ± 4.66%, corresponding to the extract P2-F-E, obtained from flowers of Population 2 with 70% ethanol, while the lowest inhibitions were present in the leaf extracts of Populations 1, 2, and 3, specifically the extract P1-L-M, obtained from leaves of Population 1 with 70% methanol, extract P3-L-E, extracted from leaves of Population 3 with 70% ethanol, and extract P2-L-M, extracted from leaves of Population 2 with 70% methanol with inhibition percentages of 61.99 ± 4.54, 61.72 ± 15.19, and 60.92 ± 5.87%, respectively. It is important to highlight that the results obtained by the extracts of the leaf and flower of *C. sulphureus* are considered relevant in terms of the ability of the extracts to inhibit lipid oxidation since, to the authors’ best knowledge, the inhibition of the lipid peroxidation of linoleic acid has not been previously reported for the *C. sulphureus* species analyzed in this work.

According to the analysis, the oxygen radical antioxidant capacity (ORAC) of the extracts from leaves and flowers of three populations of *C. sulphureus* ranged from 1.22 ± 1.14 to 4.88 ± 0.21 µM TE (Table 2). Previous research results reported a concentration of 214.8 ± 10.0 μM TE/g DW [27] and 317.91 µM TE/g FW [24] in *C. sulphureus* edible flowers extracted with acidified ethanol, higher values compared to those obtained from the extracts analyzed here. The ORAC assay measures specifically the ability of compounds to scavenge oxygen free radicals and, as such, is considered to be the closest to human physiology [35]. Although the *C. sulphureus* extracts showed a relevant oxygen radical scavenging activity, the correlation analysis revealed lower associations between the oxygen radical scavenging activities and the phenolic, flavonoid, and condensed tannin contents in the extracts.

## 4. Conclusions

The phenolic composition of leaf and flower extracts of *C. sulphureus* showed that the main compounds were flavonoids and phenolic acids. The recognized antioxidant flavonoids, rutin, and quercetin were identified in the extracts. Significant differences in the phenolic contents, both total and individual, were observed between leaf and flower extracts of *C. sulphureus*. The highest concentrations of phenolic compounds were observed in the samples extracted with 70% methanol. However, in the antioxidant activity determinations, no direct influence of the solvent on antioxidant activity was shown. *C. sulphureus* may be considered a potential source of natural antioxidants from the point of ethnopharmacological usage of this plant.

## Figures and Tables

**Table 1:** Results of the qualitative phytochemical analysis of ethanolic and methanolic leaf and flower extracts from three populations of *C. sulphureus*.

Phytochemical Constituents	70% Ethanol Extracts		70% Methanol Extracts	
	Leaf	Flower	Leaf	Flower
Alkaloids	−	−	−	−
Phenolic compounds	+	+	+	+
Tannins	+	+	+	+
Flavonoids	+	+	+	+
Glycosides	−	−	−	−
Terpenoids	+	+	+	+
Carbohydrates	+	+	+	+
Proteins	−	−	−	−
Amino acids	−	−	−	−
Saponins	−	−	−	−
Coumarins	+	−	+	−
Quinones	−	+	−	+
Anthraquinones	−	−	−	−
Sterols	−	+	−	+

+ indicates the presence of the compounds in the tissues. − indicates absence.

**Table 2:** Total phenolic, flavonoid, and tannin contents, free radical scavenging activity, ferric reducing antioxidant power, lipid peroxidation inhibition, and oxygen radical absorbance capacity of extracts from leaves and flowers of *Cosmos sulphureus*.

Extract	TPC (mg GAE/g DE)	TFC (mg QE/g DE)	CTC (mg CE/g DE)	DPPH^•^IC_50_ (mg/mL)	FRPIC_50_ (mg/mL)	LPI (%)	ORAC (µM TE)
P1-L-M	119.64 ± 5.61 ^ab^	95.22 ± 2.71 ^c^	38.38 ± 1.03 ^c^	0.196 ± 0.03 ^a^	0.54 ± 0.07 ^d^	61.99 ± 4.54 ^b^	1.48 ± 0.08 ^e^
P2-L-M	108.64 ± 2.80 ^bc^	85.50 ± 4.59 ^cde^	49.69 ± 1.36 ^b^	0.460 ± 0.02 ^bc^	0.60 ± 0.09 ^de^	60.92 ± 5.87 ^b^	3.68 ± 1.02 ^cd^
P3-L-M	102.68 ± 4.13 ^cd^	117.17 ± 2.40 ^b^	68.70 ± 7.19 ^a^	0.410 ± 0.01 ^abc^	0.74 ± 0.09 ^f^	65.98 ± 6.20 ^ab^	3.69 ± 0.25 ^cd^
P1-F-M	124.11 ± 7.77 ^a^	90.97 ± 2.26 ^cd^	10.64 ± 3.56 ^d^	1.118 ± 0.01 ^d^	0.31 ± 0.34 ^a^	74.15 ± 5.25 ^ab^	4.29 ± 0.27 ^abc^
P2-F-M	40.74 ± 2.34 ^h^	162.55 ± 9.32 ^a^	5.33 ± 1.80 ^d^	0.417 ± 0.02 ^abc^	0.37 ± 0.06 ^ab^	69.18 ± 9.97 ^ab^	1.22 ± 1.14 ^e^
P3-F-M	67.91 ± 0.199 ^f^	80.24 ± 1.22 ^cde^	8.24 ± 1.29 ^d^	0.895 ± 0.05 ^d^	0.42 ± 0.13 ^b^	70.33 ± 3.62 ^ab^	3.89 ± 0.61 ^bc^
P1-L-E	44.98 ± 3.21 ^h^	93.24 ± 2.77 ^c^	33.76 ± 2.72 ^c^	0.337 ± 0.02 ^ab^	0.51 ± 0.06 ^cd^	65.09 ± 5.35 ^ab^	3.22 ± 0.49 ^ab^
P2-L-E	69.60 ± 1.22 ^f^	91.65 ± 12.43 ^cd^	38.73 ± 1.07 ^c^	0.246 ± 0.01 ^ab^	0.63 ± 0.02 ^e^	70.96 ± 9.78 ^ab^	4.59 ± 0.23 ^a^
P3-L-E	94.61 ± 5.14 ^de^	124.04 ± 1.64 ^b^	60.99 ± 2.24 ^a^	0.348 ± 0.01 ^abc^	0.59 ± 0.05 ^de^	61.72 ± 9.12 ^b^	3.91 ± 0.58 ^bc^
P1-F-E	82.60 ± 3.97 ^e^	73.72 ± 3.23 ^e^	9.44 ± 2.59 ^d^	1.922 ± 0.29 ^e^	0.43 ± 0.14 ^bc^	74.95 ± 2.28 ^ab^	4.50 ± 0.42 ^ab^
P2-F-E	48.42 ± 2.98 ^gh^	76.75 ± 3.50 ^de^	7.90 ± 2.14 ^d^	0.601 ± 0.02 ^c^	0.30 ± 0.06 ^a^	83.83 ± 4.66 ^a^	4.25 ± 0.17 ^abc^
P3-F-E	59.52 ± 3.89 ^fg^	50.16 ± 7.65 ^f^	5.67 ± 1.65 ^d^	1.040 ± 0.04 ^d^	0.37 ± 0.20 ^ab^	79.71 ± 4.07 ^ab^	4.88 ± 0.21 ^a^

Abbreviations: TPC, total phenolic content; TFC, total flavonoid content; CTC, condensed tannin content; GAE, gallic acid equivalents; QE, quercetin equivalents; CE, catechin equivalents; DE, dry extract; DPPH^•^, 2,2-diphenyl-1-picrylhydrazyl; IC_50_, median inhibitory concentration; FRP, ferric reducing power; LPI, lipid peroxidation inhibition; ORAC, oxygen radical absorbance capacity; TE, Trolox equivalents; P1, population 1; P2, population 2; L, leaves; F, flowers; M, extracted with 70% methanol; E, extracted with 70% ethanol. Values are expressed as mean ± standard deviation of three repetitions. Values with different letters indicate significant differences (Tukey, *p* < 0.05).

**Table 3:** Phenolic compounds identified by High-Performance Thin-Layer Chromatography (HPTLC) in methanolic extracts from leaves and flowers of *Cosmos sulphureus*.

#	R_f_	Compound	Population 1		Population 2		Population 3	
			µg/mL					
			Leaf	Flower	Leaf	Flower	Leaf	Flower
1	0.48 ± 0.04	Rutin	16.98 ± 2.79 ^a^	6.95 ± 2.10 ^b^	17.24 ± 0.71 ^a^	14.09 ± 0.90 ^a^	18.96 ± 2.52 ^a^	7.89 ± 0.68 ^b^
2	0.60 ± 0.04	Chlorogenic acid	23.93 ± 10.71 ^ab^	27.51 ± 4.87 ^a^	11.16 ± 0.41 ^c^	13.25 ± 0.55 ^bc^	19.28 ± 0.90 ^abc^	9.10 ± 0.93 ^c^
3	0.71 ± 0.03	Caffeic acid	ND	146.30 ± 29.39 ^a^	ND	68.09 ± 8.83 ^b^	ND	53.11 ± 6.26 ^b^
4	0.78 ± 0.04	Quercetin	ND	309.03 ± 59.57 ^a^	ND	68.03 ± 8.80 ^b^	ND	64.97 ± 1.63 ^b^

Abbreviations: R_f_, retardation factor; ND, not determined. Values are expressed as mean ± standard deviation of three repetitions. Values with different letters indicate significant differences (Tukey, *p* < 0.05).

**Table 4:** Phenolic compounds identified by High-Performance Thin-Layer Chromatography (HPTLC) in ethanolic extracts from leaves and flowers of *Cosmos sulphureus*.

#	R_f_	Compound	Population 1		Population 2		Population 3	
			µg/mL					
			Leaf	Flower	Leaf	Flower	Leaf	Flower
1	0.48 ± 0.04	Rutin	22.48 ± 6.16 ^a^	9.74 ± 6.64 ^b^	19.23 ± 1.64 ^ab^	27.93 ± 1.90 ^a^	23.00 ± 1.22 ^a^	25.21 ± 4.17 ^a^
2	0.60 ± 0.04	Chlorogenic acid	16.29 ± 4.38 ^ab^	22.34 ± 4.03 ^ab^	11.75 ± 0.27 ^b^	19.69 ± 2.38 ^ab^	16.45 ± 0.94 ^ab^	18.43 ± 4.60 ^ab^
3	0.71 ± 0.03	Caffeic acid	ND	175.93 ± 28.42 ^a^	ND	126.30 ± 17.76 ^b^	ND	124.63 ± 10.04 ^b^
4	0.78 ± 0.04	Quercetin	ND	330.70 ± 52.98 ^a^	ND	121.23 ± 1.69 ^b^	ND	149.93 ± 1.19 ^b^

Abbreviations: R_f_, retardation factor; ND, not determined. Values are expressed as mean ± standard deviation of three repetitions. Values with different letters indicate significant differences (Tukey, *p* < 0.05).

## Data Availability

The authors declare that the data supporting the findings of this study are available within the article.
